# Wingspan Stenting for Severe Symptomatic Intracranial Atherosclerotic Stenosis in 433 Patients Treated at a Single Medical Center

**DOI:** 10.1371/journal.pone.0139377

**Published:** 2015-09-30

**Authors:** Tian-Xiao Li, Bu-Lang Gao, Dong-Yang Cai, Zi-Liang Wang, Liang-Fu Zhu, Jiang-Yu Xue, Wei-Xing Bai, Ying-Kun He, Li Li

**Affiliations:** 1 Stroke Center, Henan Provincial People’s Hospital, Zhengzhou University, Zhengzhou, Henan Province, China; 2 Department of Medical Research, Shijiazhuang First Hospital, Hebei Medical University, Shijiazhuang, Hebei Province, China; University of Münster, GERMANY

## Abstract

**Purpose:**

To investigate the safety and outcome of intracranial stenting for intracranial atherosclerotic stenosis (IAS).

**Materials and Methods:**

Between July 2007 and April 2013, 433 consecutive patients with IAS >70% underwent intracranial Wingspan stenting, and the data were prospectively analyzed.

**Results:**

Intracranial stenting was successful in 429 patients (99.1%), and the mean stenosis rate was improved from prestenting (82.3± 7.6)% to poststenting (16.6 ± 6.6)%. During the 30-day perioperative period, 29 patients (6.7%) developed stroke. The total perioperative stroke rate was significantly (P <0.01) higher in the basilar artery area than in others, whereas the hemorrhagic stroke rate was significantly (P <0.05) greater in the middle cerebral artery area than in others. The experience accumulation stage (13%) had a significantly (P <0.05) higher stroke rate than the technical maturation stage (4.8%). Clinical follow-up 6–69 months poststenting revealed ipsilateral stroke in 20 patients (5.5%). The one- and two-year cumulative stroke rates were 9.5% and 11.5%, respectively; the two-year cumulative stroke rate was significantly (P <0.05) greater in the experience accumulation stage (18.8%) than in the technical maturation stage (9.1%).

**Conclusion:**

Wingspan stenting for intracranial atherosclerotic stenosis is safe and the long-term stroke rate after stenting is low in a Chinese subpopulation.

## Introduction

Intracranial atherosclerotic stenosis (IAS) is the main cause of recurrent ischemic stroke. In Asia, nearly 33% of ischemic stroke cases are caused by IAS [1]. Currently, the primary treatments for IAS are antithrombotic agents, statins, antihypertensive agents, and lifestyle adjustments [2]. In the warfarin versus aspirin for symptomatic intracranial disease (WASID) study [3], patients with stenosis levels ranging 70%–99% had a recurrence rate above 20%. The clinical role of stenting, a supplementary therapy used in addition to drug treatment, has been demonstrated by several groups [4–7]. Nonetheless, a recent report of stenting versus aggressive medical management for preventing recurrent ischemic stroke in intracranial stenosis (SAMMPRIS) study [8,9] did not favor the use of the Wingspan stent (Stryker Neurovascular, Kalamazoo, MI, USA) for intracranial stenosis because the 30-day rate of stroke or death was 14.7% in the stenting arm but 5.8% in the medical-management arm, whereas the 1- and 2-year rates of the primary endpoint were 19.7% and 20.6%, respectively, in the stenting group but 12.6% and 14.1%, respectively, in the medical group. However, some aspects of the experimental design of the SAMMPRIS study were controversial, and the preventive effect of stenting on stroke recurrence needs to be further investigated [10–13]. The present study prospectively examined 433 cases of IAS treated with Wingspan stenting at our center to evaluate the safety and long-term clinical outcome of Wingspan stenting.

## Materials and Methods

### General information

This study was approved by the Henan Provincial People’s Hospital Ethics Committee for scientific research, and all patients signed the informed consent for stenting. Patients with IAS were prospectively enrolled for Wingspan stenting at our center between June 2007 and July 2013. The inclusion criteria were ≥70% stenosis of the intracranial arteries with symptomatic ischemic stroke or a transient ischemic attack (TIA), 18–85 years of age, length of each stenosis ≤15 mm, over24 hours from the final TIA event, and over 7 days from the final stroke. The exclusion criteria were nonatherosclerotic stenosis, emergency arterial occlusion, chronic arterial occlusion treated with balloon recanalization, unilateral extracranial artery stenosis, potential source of cardiac embolism, concurrence with intracranial pathology including tumors, aneurysms or arteriovenous malformation, and contraindication to antiplatelet treatment. A total of 433 patients were confirmed to have severe IAS (70–99%) via digital subtraction angiography (DSA) [14] and enrolled for Wingspan stenting.

### Stenting procedure

All procedures were performed by five experienced neurointerventional surgeons. For each surgeon, we assigned the period during which the first 20 patients underwent Wingspan stenting as his/her “experience accumulation stage”. Accordingly, the five surgeons performed a total of 100 cases at this stage. The remaining 333 cases were designated as the “technical maturation stage.” The procedure was performed under general anesthesia with systemic heparin administration to maintain the intraoperative clotting time at 250–300 seconds. Typically, access was achieved through the femoral artery with a long sheath to allow navigation of the guiding catheter tip to the distal end of the petrous carotid artery or the cervical vertebral artery. Three-dimensional angiography was subsequently performed to determine the optimal working angle, and then, road map was used to guide the passage of a 0.014-inch micro-guidewire through the stenosis. A Gateway^(TM)^ balloon was sized to 80%–90% of the normal parent artery diameter proximal or distal to the lesion and the balloon length was selected to match the stenosis. The balloon was then navigated via the micro-guidewire to the stented segment, positioned properly, and slowly filled with 50% contrast agent to a pressure of 6 atm for 10–20 s for observation of the improvement in the stenosis. After a Wingspan stent was sized to exceed the diameter of the normal parent artery by 0.5–1.0 mm and the stent length to completely cover the entire stenotic segment, it was deployed at the stenosis site. Angiography was performed to check the residual stenosis and distal vessel branches. Once the residual stenosis was not greater than 30% and the distal vessels displayed no occlusion, the micro-guidewire and guide catheter were withdrawn to end the procedure.

### Perioperative management

Before the procedure, all patients had oral antiplatelet drugs clopidogrel (75 mg/d) and aspirin (100 mg/d) for 3–5 days. In emergency cases, clopidogrel and aspirin would be administered once with a loading dose of 300 mg for each drug within 24 h prior to stenting. Nimodipine was intravenously applied 2 h before stenting. Immediately after stenting, computed tomography (CT) was performed to exclude cerebral hemorrhaging. Nimodipine was continued for 1–3 days to maintain the lower limit of basal blood pressure. Once the hemorrhage risk had been eliminated, low-molecular-weight heparin (4000–6000 U/12 h) would be used for the patient subcutaneously for 3 days. Following stenting, the dual antiplatelet regimen would be continued for 6 months before switching to aspirin alone at a dosage of 100 mg/d.

### Clinical and imaging follow-up

The incidences of stroke, death, and TIA during the 30-day perioperative period were monitored. Each patient was assessed with the modified Rankin Scale (mRS) and National Institutes of Health stroke scale (NIHSS) scores before and after stenting and at follow-ups. Clinical neurological assessments were performed at 1, 3, 6 and 12 months, and once annually afterwards. Patients with suspected recurrent strokewould have CT or magnetic resonance imaging (MRI) examination. Imaging follow-up was performed 6 months after the procedure for detecting possible in-stent restenosis (ISR) which was defined as the degree of stenosis >50% compared with presenting baseline at a site within the stent or within 5 mm immediately adjacent to the stent and the degree of stenosis >20% of absolute luminal loss since stenting [15].

### Statistical methods

Continuous variables were expressed as mean±SD and classification data were expressed as percentages. Inter-group comparisons of the data were determined with Student’s t-test, Chi-square test or Fisher's exact test if appropriate. Estimations of the cumulative stroke incidence were assessed according to the Kaplan-Meier method, and inter-group comparisons of these data were analyzed using the log-rank test. Multiple regression analysis was also performed. The P-values were all bilateral, and a P-value <0.05 was considered statistically significant.

## Results

### Baseline demography and stenting procedure

The stenting procedure was performed 23.7 ± 17.3 days after the final ischemic event (Tables 1 and 2), and all patients were hospitalized for surveillance at least 7 days after stenting. The Wingspan stent was successful in 429 patients (99.1%), and the mean stenosis rate was improved from presenting (82.3 ± 7.6)% to poststenting (16.6 ± 6.6)% (Table 2). Four patients with combined basilar and intracranial vertebral artery stenosis and another two patients with severe bilateral intracranial vertebral artery stenosis were all successfully treated with the Wingspan stenting, without perioperative complications. The four patients who failed the stenting procedure all had the target lesion in the middle cerebra artery. Of these, one patient had the procedure terminated prematurely because of minor bleeding caused by a micro-guidewire passing through the lesion, whereas the remaining three patients had extremely tortuous carotid artery siphon segment, leading to exceptional difficulty navigating the stent. Among these three patients, one underwent balloon dilatation prior to implantation of a Neuroform stent (Stryker Neurovascular, Boston, MA, USA), and the other two had balloon dilatation alone.

**Table 1:** Baseline information of the 433 patients.

Age (years)	57.3 ± 11.6
Male	299 (69.1%)
Hypertension	295 (68.1%)
Diabetes	125 (28.9%)
Hyperlipidemia	176 (40.6%)
Smoking	147 (33.9%)
History of coronary heart disease	52 (12.0%)
Previous history of stroke	102 (23.6%)
Gap between final event and stenting (days)	23.7 ± 17.3
Preoperative ischemic events (stroke)	203 (46.9%)
Preoperative mRS	
0	175 (40.4%)
1	117 (27.0%)
2	79 (18.2%)
3	62 (14.3%)

Note: mRS, modified Rankin scale.

**Table 2:** Lesion characteristics.

Variables	Data
Lesion length (mean, mm)	8.8 ± 2.4
≥10 mm	88 (20.3%)
5–10 mm	309 (71.4%)
<5 mm	36 (8.3%)
Lesion location	
Carotid artery	58 (13.4%)
Middle cerebral artery	196 (45.3%)
Vertebral artery	88 (20.3%)
Basilar artery	91 (21.0%)
Preoperative stenosis level (%)	82.3 ± 7.6
Residual stenosis after stenting (%)	16.6 ± 6.6

### Perioperative complications

**Fig 1:**
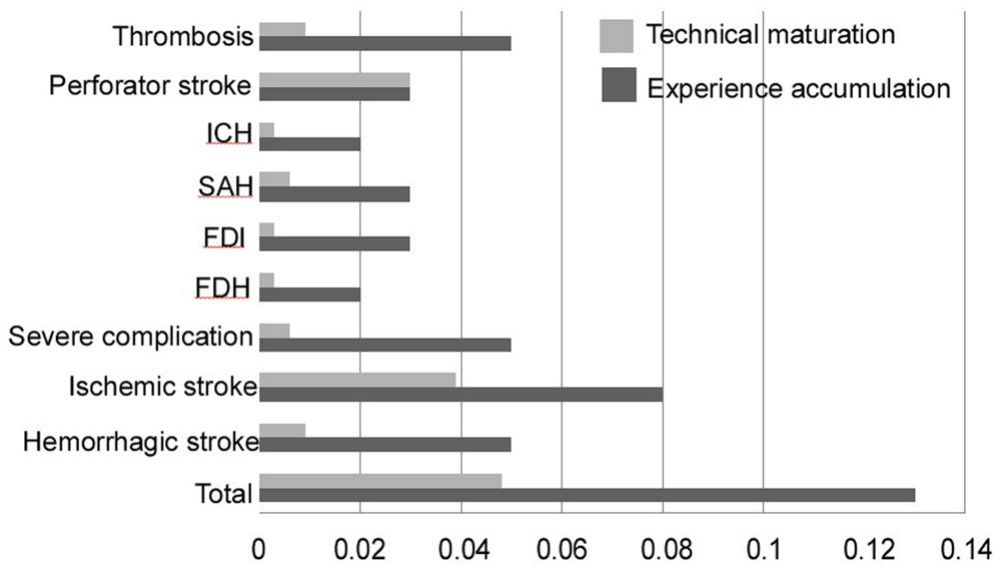
Perioperative complications with respect to the experience accumulation and technical maturation stages. ICH, intracerebral hemorrhage; SAH, subarachnoid hemorrhage; FDI, fatal/disabling ischemic stroke; FDH, fatal/disabling hemorrhage.

During the 30-day perioperative period, 29 patients (6.7%; 29/433) developed stroke or died, including 21 cases (4.8%) of ischemic stroke and eight (1.8%) hemorrhagic stroke. In addition, there were seven patients (1.6%) of fatal or disabling stroke, including three cases of death and four severe disability (Tables 3 and 4 and Fig 1). The perioperative stroke incidence was significantly greater in the experience accumulation stage than in the technical maturation stage (13% vs. 4.8%; p = 0.007). Specifically, the rates of hemorrhagic stroke, thrombotic stroke, ischemic and fatal/disabling stroke, and total fatal/disabling stroke were all significantly higher in the first stage than in the second stage (p = 0.018, p = 0.018, p = 0.04, and p = 0.008, respectively). The incidence of perforator stroke did not differ between the two stages (3% vs. 3%; p = 1.000).

**Table 3:** The 30-day perioperative complications with respect to different stages.

Events	N	Experience accumulation stage (n = 100)	Technical maturation stage (n = 333)	P
Hemorrhagic stroke	8	5 (5%)	3 (0.9%)	0.018
Fatal/disabling	3 (2 dead)	2	1	0.135
Non-fatal/disabling	5	3	2	0.083
SAH	5	3	2	0.083
ICH	3	2	1	0.135
Ischemic stroke	21	8 (8%)	13 (3.9%)	0.111
Fatal/disabling	4 (1 dead)	3	1	0.04
Non-fatal/disabling	17	5	12	0.558
Thrombosis	8	5 (5%)	3 (0.9%)	0.018
Perforator stroke	13	3 (3%)	10 (3%)	1.000
Severe complications	7	5 (5%)	2 (0.6%)	0.008
Total	29	13 (13%)	16 (4.8%)	0.007

Note: The SAH was considered technically related in all 5 patients while the reason for ICH was not definitive in all 3 patients. SAH, subarachnoid hemorrhage; ICH, intracerebral hemorrhage.

**Table 4:** The 30-day perioperative complications with respect to the lesion sites.

Events	N	Basilar N = 91	MCA N = 196	VA N = 88	ICA N = 58	P
Hemorrhagic stroke	8	1 (1.1%)	7 (3.6%)	0	0	0.026^b^
Non-fatal/disabling	3	0	3 (1.5%)	0	0	0.092^b^
SAH	5	1 (1.1%)	4 (2.0%)	0	0	0.181^b^
ICH	3	0	3 (1.5%)	0	0	0.092^b^
Ischemic stroke	21	12 (13.2%)	7 (3.6%)	1 (1.1%)	1 (1.7%)	0.000^a^
Fatal/disabling	4	2 (2.2%)	2 (1.0%)	0	0	0.196^a^
Non-fatal/disabling	8	4 (4.4%)	2 (1.0%)	1 (1.1%)	1 (1.7%)	0.064^a^
Thrombosis	13	8 (8.8%)	5 (2.6%)	0	0	0.001^a^
Perforator stroke	7	2 (2.2%)	5 (2.6%)	0	0	0.641^a^
Severe complications	29	13 (14.3%)	14 (7.1%)	1 (1.1%)	1 (1.7%)	0.002^a^

Note: MCA, middle cerebral artery; VA, vertebral artery; ICA, internal carotid artery; SAH, subarachnoid hemorrhage; ICH, intracerebral hemorrhage.

^a:^ Comparison of basilar artery to other sites, and

^b:^ Comparison of middle cerebral artery to other sites.

Compared with other sites (e.g., middle cerebral artery, intracranial vertebral and internal carotid arteries) in the brain, the basilar artery exhibited a higher incidence of nervous system complications during the 30-day perioperative period (14.3% vs. 4.7%; p = 0.002). This result was clearly demonstrated by the significantly higher incidences of ischemic stroke and perforator stroke (p = 0.000 and p = 0.001, respectively). However, none of the perioperative perforator stroke cases in the basilar artery were fatal or disabling. The incidence of hemorrhagic stroke in the 30-day perioperative period was significantly higher in the middle cerebral artery than at the other sites (3.6% vs. 0.4%; p = 0.026).

### Clinical follow-up results

**Fig 2:**
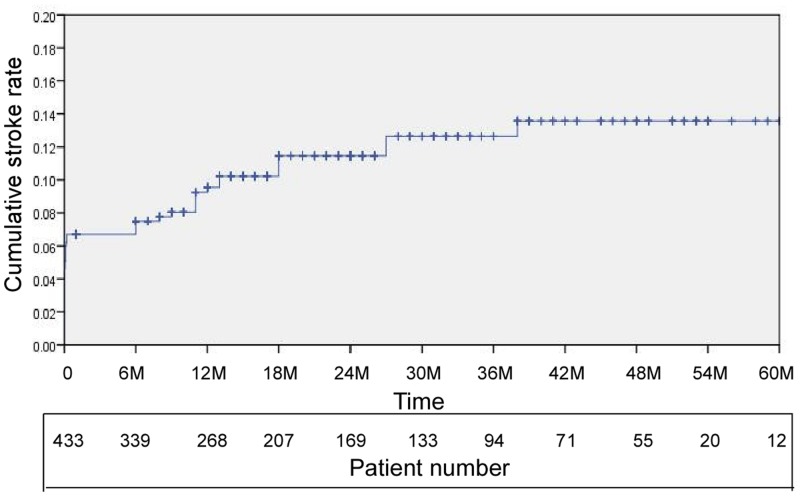
Kaplan-Meier estimation of the cumulative stroke rate. The analysis ended at 60 months (x-axis) because only two cases had a follow-up duration exceeding 66 months. The one-year cumulative stroke rate (including all cases of stroke or death within the 30-day perioperative period and all cases of ipsilateral stroke after that period) was 9.5% (95% CI: 6.6–12.4%) and the two-year cumulative stroke rate was 11.5% (95% CI: 8.2%–14.8%).

A total of 365 patients (84.3%; 365/433) were followed up at a mean duration of 29.6 ± 16.3 months (range: 6–69 months). After the 30-day perioperative period, 20 patients (5.5%; 20/366) developed ipsilateral stroke, including one case of fatal stroke and one case of disabling stroke (0.5%; 2/365). Seventeen patients (4.7%; 17/365) suffered TIA near the treated blood vessels. Moreover, four patients (two of whom died) developed stroke in areas outside the treated blood vessels, two suffered cerebral hemorrhages and died, and another five died from non-neurological diseases. The Kaplan-Meier survival analysis yielded a one-year cumulative stroke rate (including all cases of stroke or death within the 30-day perioperative period and all cases of ipsilateral stroke after this period) of 9.5 (95% CI: 6.6–12.4%) and a two-year cumulative stroke rate of 11.5% (95% CI: 8.2–14.8%) (Fig 2).

**Fig 3:**
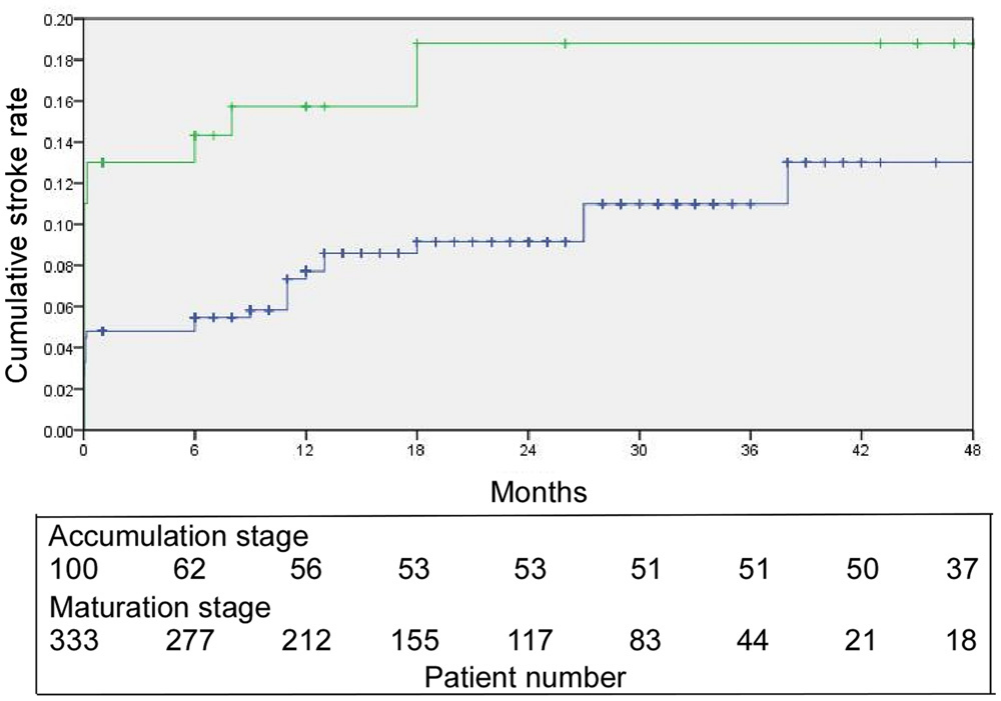
Kaplan-Meier estimation of the cumulative stroke rate. The stroke incidences during the experience accumulation and technical maturation stages have been compared. The former stage yielded a one-year cumulative stroke rate (including all cases of stroke or death within the 30-day perioperative period and all cases of ipsilateral stroke after that period) of 15.7% (95% CI: 8.3–23.1%) and a two-year cumulative stroke rate of 18.8% (95% CI: 10.6–27.0%). The latter stage generated a one-year cumulative stroke rate (including all cases of stroke or death within the 30-day perioperative period and all cases of ipsilateral stroke after that period) of 7.7% (95% CI: 4.8–10.6%) and a two-year cumulative stroke rate of 9.1% (95% CI: 5.8–12.4%). The Kaplan-Meier plots differed significantly according to the log-rank test (*X* ^2^ = 4.735, p = 0.030). Green curve: Experience accumulation stage. Blue curve: Technical maturation stage.

The experience accumulation stage had a one-year cumulative stroke rate (including all cases of stroke or death within the 30-day perioperative period and all cases of ipsilateral stroke after this period) of 15.7% (95% CI: 8.3–23.1%) and a two-year cumulative stroke rate of 18.8% (95% CI: 10.6–27.0%) (Fig 3). In comparison, the technical maturation stage had a one-year cumulative stroke rate (including all cases of stroke or death within the 30-day perioperative period and all cases of ipsilateral stroke after this period) of 7.7% (95% CI: 4.8–10.6%) and a two-year cumulative stroke rate of 9.1% (95% CI: 5.8–12.4%). The Kaplan-Meier curves indicated significant differences according to the log-rank test (χ^2^ = 4.735, p = 0.030).

### Multivariable analysis

A multivariable analysis investigated the following factors in affecting the stroke events during the perioperative period and at follow-up including sex, age, presenting symptoms, time from symptom onset, stenting experience, prestenting hypertension, diabetes mellitus, hyperlipemia, hemocyanin, coronary heart disease, smoking history, prestenting mRS, prestenting stenosis extent and length, stenosis location and follow-up duration. During the 30-day perioperative period, the events of total complications, thrombus formation, and ischemia were all significantly affected by the technical experience of stenting (P < 0.001), smoking history (P < 0.05), stenosis location (P < 0.05) and length (P = 0.0038), and time of event onset (P < 0.001). However, the total post-stenting bleeding events, intracerebral hemorrhage and subarachnoid hemorrhage were all significantly affected by the technical experience of stenting (P < 0.01), prestenting stenosis extent (P < 0.05) and length (P < 0.05), and time of event onset (P < 0.01). The subarachnoid hemorrhage was also significantly affected by hyperlipemia (P = 0.046) and presenting mRS (P = 0.0197). For the total period immediately after stenting up to two years of follow-up, the total stroke events were significantly affected by hyperlipemia (P = 0.0187), smoking history (P = 0.0103), post-stenting residual stenosis (P = 0.0099) and total perioperative complications (P < 0.0001).

### Imaging results

Imaging follow-up was performed on 208 patients 3–53 (mean 11.0 ± 9.2) months after stenting, including CT scanning in 40 and regular DSA in 168 patients. Sixty-two patients (29.8%, 62/208) had in-stent restenosis, including symptomatic restenosis in 18 (8.7%; 18/208). Twelve of the 18 symptomatic patients subsequently received secondary treatment (stent deployment in two cases and simple balloon dilatation in the rest) with no procedural complications.

## Discussion

Traditionally, stenting was used only as a complementary therapy to anticoagulation in patients with severe symptomatic IAS who, despite standard medications, exhibited recurrent stroke or apparent blood hypoperfusion in areas affected by the stenosis [3,16]. Whether stenting can prevent ischemic stroke is controversial [8,9,17], and long-term therapeutic effect of stenting thus still requires verification [11–13].

The study by the SAMMPRIS group [8] suggested that perioperative complications were not associated with the surgeons’ technical experience because no significant difference (P >0.05) was found in the complication rates between high- and low-enrolling sites and the 30-day stroke and death rates did not increase along with increase of enrolled patients. Nonetheless, our study revealed a significantly higher (P <0.05) complication rate during the experience accumulation stage than the technical maturation stage. A detailed analysis further demonstrated that the incidences of hemorrhagic, thrombotic, and fatal/disabling complications were closely related to the surgeons’ experience levels, and the rates of complications pertinent to surgical techniques exhibited an apparent learning curve, consistent with other studies [18,19].

Stenting-related ischemic complications were more likely in the posterior than the anterior circulation [20–22]. Moreover, the “snow-plow effect” resulting from stent or balloon squeezing and debris displacement might be the main cause of posterior perioperative perforator strokes during stenting [20]. This is because the middle portion of the basilar artery contains many small perforating vessels, and stenting-induced debris displacement may completely clog these perforators, consequently causing small infarction and minor disabilities. This does not involve technical experience as proved by our study in which the posterior perioperative perforator stroke incidence, unlike at other sites, did not decrease with accumulated experience. Hemorrhagic complications were more frequent in the middle cerebral artery territory than others [21], and the increased bleeding propensity in this area was probably related to the tortuous carotid artery siphon, which makes it difficult for endovascular devices to pass through, leading to possible vascular abrasion.

The long-term stroke incidence of symptomatic IAS is related to the stenosis degree. Some studies [3,23] reported a 19% stroke incidence in regions near the treated arteries with a >70% stenosis during follow-up, markedly higher than with a <70% stenosis. In theory, patients with a >70% stenosis can benefit from stenting, despite high rates of perioperative complications from stenting [5,18]. Recently, some reports [8–10] described higher perioperative stroke rates in patients with 70%–99% stenosis treated with stenting or drugs, and follow-up revealed an approximate stroke incidence of 10% in both groups, indicating that stenting had no superior ischemia preventive effect in comparison with medications. However, the present study presented a different but superior picture of stenting, with an ipsilateral stroke recurrence rate of only 5.5%, one- and two-year cumulative stroke rates of 9.5% and 11.5%, respectively. Consistent with the notion in other studies that insufficient stenting experience could increase the perioperative stroke rate [10,11], our study demonstrated a significant (P <0.05) influence of the surgeon’s stenting experience on the perioperative complication as well as the cumulative stroke rates at follow-up. Perioperative complications constitute a key factor influencing the overall efficacy of stenting. In dual antiplatelet therapy, strict control of the vascular risk factors could enhance the antithrombotic effect [24], but given the particular economic and cultural characteristics in China, it would be impractical to perform strict medical treatment and atherosclerosis risk factor control in these patients. In China, the great majority of population do not have health insurance, which limits long-term prophylactic application of antiplatelet medications. Moreover, the Chinese methods of cooking and daily dieting also make it difficult to adhere to long-time use of medications for atherosclerotic risk control. Thus, a similar outcome to that of the SAMMPRIS study [8–10] cannot be achieved with medication alone, and stenting appears to be a better choice for Chinese patients with IAS.

A multivariable analysis in this study proved that the technical experience of stenting did significantly (P < 0.05) affect the total 30-day perioperative stroke events, including both ischemic and bleeding events. The ischemic events were also significantly (P < 0.05) affected by smoking history, stenosis location and length, whereas the bleeding events by stenosis extent and length. For the stroke events after stenting up to two years of follow-up, the significantly (P < 0.05) affecting factors included hyperlipemia, smoking history, post-stenting residual stenosis, and total perioperative complications. Since the total perioperative complications were significantly (P<0.01) affected by the technical experience of stenting, the total stroke events for two years of follow-up were also significantly affected by the technical experience of stenting.

In the SAMMPRIS study, the incidence of fatal/disabling stroke during follow-up was very low in patients with stenting compared with patients treated with medication alone (2.6%, 5/191 vs. 6.7%, 14/210; p = 0.046) (Table 5). In our study, only two of the 365 stented patients (0.5%) developed fatal/disabling stroke despite aggressive medications, indicating that aggressive medical management might sufficiently reduce the long-term risks of death and disability in stented patients. Moreover, the complication rate was significantly lower (P < 0.05) at the technical maturation stage than at the initial experience accumulation stage, and with accumulated experience, stenting could increasingly become a safe and effective approach for IAS.

**Table 5:** Disabling or fatal stroke rate within and beyond 30 days in the SAMMPRIS study.

Groups (n)	30-day Disabling/fatal stroke rate (n)	Disabling/fatal stroke rate during follow-up (n)	Disabling/fatal stroke rate beyond 30 days
PTAS (224)	7.0% (16)#	9% (21) *	2.6% (5/191) **
MED (227)	1.8% (4)#	8% (18) *	6.7% (14/210) **

Note:

^#^ The disabling or fatal stroke rates within 30 days could be found in Table 3 in reference 8.

* The disabling or fatal stroke rates at the end of 32.4 months of follow-up for both groups could be found in Table 3 in reference 9.

** The number of patients with disabling or fatal stroke beyond 30 days was 21–16 = 5 for the PTAS group but 18–4 = 14 for the MED group, whereas the number of patients who finished the follow-up was 191 for the PTAS group and 210 for the MED group (see the Results of reference 9). PTAS: percutaneous transluminal angioplasty and stenting; MED: medical management group.

This study has some limitations. Besides none randomization and none control, only Chinese patients were enrolled at a single high-volume center. Another limitation is the single center design of study. Moreover, 15.7% of the stented patients were lost to follow-up, which may be caused primarily by the following fact that most people do not have health insurance in China and that they will not come for a clinical follow-up if they do not have stroke symptoms. Even so, it may affect the estimates of long-term stroke free outcome. In the future, a randomized, controlled study with rigorous follow-up protocol involving multiple high-volume centers or different stents will be needed to investigate whether stenting in combination with intensive medical management is superior to aggressive medications alone for IAS.

In conclusion, the Wingspan stenting for intracranial atherosclerotic stenosis is safe in this study and, in combination with aggressive medication management, may hopefully improve intracranial atherosclerotic stenosis and the long-term stroke rate.

**Key Points**

1. The surgeons’ technical experience does influence the long-term complications of intracranial stenting.
2. Intracranial stenting can decrease long-term risk of death and disability.
3. Stenting can significantly improve intracranial atherosclerotic stenosis with a long-lasting effect.
4. Stenting is no longer a complementary therapy for intracranial atherosclerotic stenosis.
